# Does Self-Efficacy Affect Cognitive Performance in Persons with Clinically Isolated Syndrome and Early Relapsing Remitting Multiple Sclerosis?

**DOI:** 10.1155/2015/960282

**Published:** 2015-04-29

**Authors:** Peter Joseph Jongen, Keith Wesnes, Björn van Geel, Paul Pop, Hans Schrijver, Leo H. Visser, H. Jacobus Gilhuis, Ludovicus G. Sinnige, Augustina M. Brands

**Affiliations:** ^1^MS4 Research Institute, Ubbergseweg 34, 6522 KJ Nijmegen, Netherlands; ^2^Department of Community & Occupational Medicine, University Medical Center Groningen, Antonius Deusinglaan 1, 9713 AV Groningen, Netherlands; ^3^Swinburne University, John Street, Hawthorn, Melbourne, VIC 3122, Australia; ^4^Medisch Centrum Alkmaar, Wilhelminalaan 12, 1815 JD Alkmaar, Netherlands; ^5^Viecuri Medisch Centrum, Merseloseweg 130, 5801 CE Venray, Netherlands; ^6^Westfries Gasthuis, Maelsonstraat 3, 1624 NP Hoorn, Netherlands; ^7^St. Elisabeth Ziekenhuis, Hilvarenbeekseweg 60, 5022 GC Tilburg, Netherlands; ^8^Reinier de Graaf Gasthuis, Reinier de Graafweg 3-11, 2625 AD Delft, Netherlands; ^9^Medisch Centrum Leeuwarden, Henri Dunantweg 2, 8934 AD Leeuwarden, Netherlands; ^10^Department of Experimental Psychology, Utrecht University, Heidelberglaan 2, 3584 CS Utrecht, Netherlands; ^11^Zuwe Hofpoort Ziekenhuis, Regionaal Psychiatrisch Centrum Woerden, Polanerbaan 2, 3447 GN Woerden, Netherlands

## Abstract

In persons with multiple sclerosis (MS) a lowered self-efficacy negatively affects physical activities. Against this background we studied the relationship between self-efficacy and cognitive performance in the early stages of MS. Thirty-three patients with Clinically Isolated Syndrome (CIS) and early Relapsing Remitting MS (eRRMS) were assessed for self-efficacy (MSSES-18), cognition (CDR System), fatigue (MFIS-5), depressive symptoms (BDI), disease impact (MSIS-29), and disability (EDSS). Correlative analyses were performed between self-efficacy and cognitive scores, and stepwise regression analyses identified predictors of cognition and self-efficacy. Good correlations existed between total self-efficacy and Power of Attention (*r*= 0.65; *P*< 0.001), Reaction Time Variability (*r*= 0.57; *P*< 0.001), and Speed of Memory (*r*= 0.53; *P*< 0.01), and between control self-efficacy and Reaction Time Variability (*r*= 0.55; *P*< 0.01). Total self-efficacy predicted 40% of Power of Attention, 34% of Reaction Time Variability, and 40% of Speed of Memory variabilities. Disease impact predicted 65% of total self-efficacy and 58% of control self-efficacy variabilities. The findings may suggest that in persons with CIS and eRRMS self-efficacy may positively affect cognitive performance and that prevention of disease activity may preserve self-efficacy.

## 1. Introduction

Multiple sclerosis (MS) is a chronic inflammatory and degenerative disease of the central nervous system (CNS), for which no definite cure is available. In over 80% of the persons with MS (PwMS) the initial phase of the disease is characterized by relapses, which are typically followed by a complete or partial recovery (Relapsing Remitting MS, RRMS). The frequency and severity of relapses are largely unpredictable but may be reduced by disease modifying drugs (DMDs). Treatment of Clinically Isolated Syndrome (CIS) suggestive of MS with a DMD may delay the conversion to RRMS [1], and DMD treatment of RRMS may substantially decrease the risk of conversion to secondary progressive MS [2, 3].

Self-efficacy is a core concept of social cognitive theory [4–6] that refers to the degree in which a person is confident to complete tasks and to reach goals in specific situations [4–6]. It is influenced by experience, social persuasion, and physiological factors [4, 6]. Self-efficacy itself may affect human function in various ways. First, by influencing choices regarding behavior, people generally avoid tasks where self-efficacy is low but undertake tasks where self-efficacy is high [7]. Second, by affecting motivation, people with high self-efficacy are more likely to make efforts to complete a task and to persist longer in those efforts, whereas those with low self-efficacy will tend toward discouragement and giving up [7, 8]. Third, self-efficacy has effects on thought patterns and responses: low self-efficacy can lead people to believe tasks to be harder than they actually are, which often results in poor planning and increased stress [7].

Self-efficacy is one of the most consistent determinants of physical activity across populations, including those with MS [9]. It has been known that MS is associated with a large reduction in physical activity behaviors, and evidence indicates that this reduction correlates negatively with self-efficacy [10]. Thus, it has been demonstrated that in PwMS self-efficacy has a positive relationship with physical activity [11–13], physical and psychological health-related quality of life (HRQoL) [14–16], and psychological adjustment [17] and is negatively associated with depression [8].

Cognitive impairment is a disabling symptom in MS, occurring in 45–65% of the patients [18]. It involves complex attention, information processing speed, (episodic) memory, and executive functions [19] and has a major impact on vocational status, interpersonal relationships, and HRQoL. Interestingly, it is not known whether in PwMS, similar to the association between self-efficacy and physical activities, self-efficacy is positively related to cognitive performance. Recently, Paunonen and Hong evaluated in university students the contribution of self-efficacy to task performance in specific cognitive ability domains and found that beliefs about verbal, numerical, and spatial capabilities correlated well with the actual performance on standardized tests [20]. So, we hypothesized self-efficacy to be a determinant of cognitive performance in PwMS. If this is the case, then interventions aimed at increasing self-efficacy could be thought of to improve cognitive abilities in this patient group, potentially most effectively in the early phases of the disease.

Therefore we analyzed the relationship between self-efficacy and cognitive performance in persons with CIS and early RRMS (eRRMS) and expected self-efficacy to positively correlate with cognitive domain performances. The investigation was part of the Cognition and Socio-Economics (COGNISEC) study, in which the associations between cognition and socioeconomics are being assessed in these patient groups [21]. Baseline findings on the relationship between working hours and power of attention, memory, fatigue, depression, and self-efficacy have been published [21].

## 2. Materials and Methods

The COGNISEC study is a prospective observational multicentre study in Netherlands on the relationship between cognition and socioeconomics in persons with CIS and eRRMS [21]. The study protocol was submitted to the ethics committee Independent Review Board (IRB). The IRB concluded that because of the observational design of the study a formal review was not required. The study is being carried out in compliance with the Declaration of Helsinki.

Patients were recruited in the outpatient departments of seven general hospitals. Patients who agreed to participate signed an informed consent form. The inclusion criteria were (1) diagnosis CIS or RRMS according to the revised McDonald criteria [22], (2) maximum time since diagnosis of two years, (3) maximum duration of DMD treatment, if any, of six months, (4) no relapse, (5) clinical stability for at least 30 days, and (6) written informed consent. The exclusion criteria were (1) worsening of symptoms suggestive of a relapse, (2) diagnosis CIS or RRMS for more than two years, (3) DMD treatment longer than six months, and (4) progressive MS. The first patient was included on February 16, 2010, and the last patient on January 5, 2012. Primary outcomes of the present analysis were the correlations between self-efficacy and cognitive domain scores at study baseline.

### 2.1. Assessment of Self-Efficacy

Self-efficacy was assessed by the Multiple Sclerosis Self-Efficacy Scale-18 (MSSES-18). The MSSES-18 is an 18-item psychometrically validated self-report questionnaire for the assessment of self-efficacy in PwMS [23]. The MSSES-18 consists of two 9-item subscales of Function and Control. Each item is scored on a Likert-like scale from 10 (very uncertain) to 100 (very certain). The MSSES-Function, MSSES-Control, and MSSES-Total scores are obtained by addition of the respective item scores and presented as percentages of the maximum scores. The MSSES-Function subscale measures confidence with functional abilities, whereas the MSSES-Control subscale measures confidence with managing symptoms and coping with the demands of illness [23].

### 2.2. Assessment of Cognition

Cognition was assessed by the CDR System, a brief, multiple repeatable, computerized battery of cognitive tests, that has been validated in various disease states and cognitive disorders including dementia, epilepsy, sleep disorders, and RRMS [24–26]. The battery uses alternate forms of tests for each testing occasion and randomizes these across repeated assessments. The forms are conceptually equivalent and the use of randomization prevents systematic bias in comparison between visits when comparing between or within groups. To minimize the motor requirement in responding, patient responses are recorded via a simple response box with two large buttons, one marked “YES” and one marked “NO” in the patient's own language. The patient is not required to use the computer keyboard or mouse and in the word recall tests oral responses are recorded by the test administrator. The tests were administered by the MS nurses of the participating sites.

The CDR System is modular, and the selected battery measured attention and psychomotor/information processing speed (simple reaction time, choice reaction time, and digit vigilance tasks, both accuracy of responding and reaction time to visual stimulus presentation), verbal and visuospatial working memory (numeric and spatial working memory tasks), and verbal and visual episodic recognition memory [26]. It took around 15 to 20 minutes to complete the selected tests.

Seven domain scores derived from the test measures: Power of Attention, Continuity of Attention, Working Memory, Episodic Memory, Speed of Memory, Cognitive Reaction Time, and Reaction Time Variability: Power of Attention, a measure of focused attention and psychomotor/information processing speed, created from the sum of reaction times from simple reaction time, choice reaction time, and digit vigilance tasks (ms); Continuity of Attention, a measure of sustained attention, summing accuracy, and error measures from the choice reaction time and digit vigilance tasks (#); Cognitive Reaction Time, a measure of central information processing speed, created by subtracting simple from choice reaction time (ms); Reaction Time Variability, a measure of momentary fluctuations in attention, formed by summing the coefficients of variance of the three reactions times (CV); Working Memory, summing accuracy measures from the numeric and spatial working memory tasks (sensitivity index (SI)); Episodic Memory, summing accuracy measures from word recognition and picture recognition tasks (SI); and Speed of Memory, a measure of complex information processing speed, summing reaction times from the numeric and spatial working memory and word and picture recognition tasks (ms). Normative data were obtained from healthy age-matched (23–55 years) volunteers, using the CDR System normative database, formed from data gathered in a series of prior clinical trials. A validation study of the CDR System in RRMS patients demonstrated the test-retest reliability over repeated assessments and significant correlations between domain scores and the Digit Symbol Substitution Test, the Paced Auditory Serial Addition Test, the Multiple Sclerosis Functional Composite, and the Expanded Disability Status Scale (EDSS) [26].

### 2.3. Assessment of Fatigue, Depressive Symptoms, Impact of Disease, and Disability

Fatigue, depressive symptoms, impact of disease, and disability may negatively affect self-efficacy or cognition in PwMS and may thus influence the relationship between self-efficacy and cognition. Therefore these variables were measured.

Fatigue was assessed by the Modified Fatigue Impact Scale 5-Item Version (MFIS-5) [27, 28], depressive symptoms were measured with the Beck Depression Inventory (BDI) [29, 30], and the impact of disease was assessed by the Multiple Sclerosis Impact Scale-29 (MSIS-29) [31, 32].

Disability was measured with the EDSS [33], a measure widely used in MS clinical studies [34].

### 2.4. Statistical Analyses

Pearson coefficients were calculated for the correlations between the MSSES-Function, MSSES-Control, and MSSES-Total scores and the CDR System cognitive domain scores and for the interrelations between domain scores. Analyses of covariance with age as a covariate were used to compare the cognitive scores in patients to those in normal controls, and Cohen's *d* effect sizes were calculated for impairments compared to controls. Stepwise regression analyses were performed to identify the predictors of the cognitive domain scores using the MSSES, MFIS-5, BDI, MSIS-29, and EDSS scores, gender, and age. A second set of stepwise regressions was done to predict the MSSES scores with the cognitive domain, MFIS-5, BDI, MSIS-29, and EDSS scores, gender, and age as explanatory variables. We adopted the standard alpha for variables to enter the regression model of *P* < 0.15. The order of entering the variables was determined by the strength of the correlation with the outcome variable. All statistical analyses were performed using the SAS Version 9.2. *P* values < 0.05 were considered statistically significant. In line with the exploratory nature of the study no adjustments for multiple comparisons were made.

## 3. Results

### 3.1. Study Population

The COGNISEC study population (*N* = 33) was recruited in the neurological outpatient departments of seven general hospitals. The characteristics have been described [21]. In brief, the female-to-male ratio was 3.1 : 1, and mean (standard deviation (SD)) age was 39.8 (8.5) years, mean (SD) time since diagnosis 13.5 (4.8) months, and mean EDSS score 1.31 (1.10).

### 3.2. Self-Efficacy Scores

The mean (SD) and minimum-maximum values for the MSSES-Function score in the total group were 92.6 (13.2) and 31–100, for the MSSES-Control score 70.5 (18.8) and 15–100, and for the MSSES-Total score 163.1 (26.6) and 84–200. The differences between females and males were not statistically significant (*P* values 0.28 to 0.44), neither were the differences between the CIS and eRRMS subgroups (*P* values 0.26 to 0.96).

### 3.3. Cognitive Domain Scores and Intercorrelations

The values for Continuity of Attention (LSmeans [standard error], 92.1 [1]), Reaction Time Variability (48.0 [2.1]), Working Memory (1.93 [0.05]), and Episodic Memory (1.5 [0.06]) in the persons with CIS and eRRMS did not differ from age-matched normative data (*N* = 1, 409) (all *P* values > 0.05). In contrast, the Power of Attention (1181 [17.8]), Cognitive Reaction Time (192 [7.4]), and Speed of Memory (3813 [104]) values were impaired compared to the age-matched controls (1068 [1.8], *P* < 0.0001; 168 [0.8], *P* < 0.01; 3206 [17], *P* < 0.0001, resp.). Cohen's *d* effect sizes of these impairments were 1.13 for Power of Attention, 0.58 for Cognitive Reaction Time, and 1.04 for Speed of Memory.

The Pearson coefficients for the intercorrelations between the cognitive domain scores in persons with CIS and eRRMS and in controls are shown in Tables 1(a) and 1(b), respectively.

Differences between patients and controls were seen in the relationships of Power of Attention and Continuity of Attention with Episodic Memory, where both correlated in persons with CIS and eRRMS to notably greater extents than in controls. In addition, Speed of Memory correlated with Continuity of Attention and Episodic Memory to a greater extent in patients than controls.

### 3.4. Correlations between Self-Efficacy and Cognitive Scores

The Pearson coefficients for the correlations between the MSSES scores and cognitive domain scores are presented in Table 2.

Of the 21 correlations explored, seven had an *r* value ≥0.49 and three an *r* value ≥0.55. Eight correlations showed a *P* value <0.01 and two a *P* value <0.001. All cognitive domain scores, except for Working Memory, correlated with function self-efficacy, control self-efficacy, or both. When we considered only correlations with *P* value <0.01, we observed that function self-efficacy correlated with attention measures and control self-efficacy with reaction time measures and that both self-efficacy measures correlated with Speed of Memory. Moreover, when selecting only correlations with *P* value <0.001, we saw that total self-efficacy correlated strongly with Power of Attention (*r* = 0.65) and Reaction Time Variability (*r* = 0.57).

### 3.5. Multivariate Predictions

Results of stepwise regression analysis to identify predictors of the cognitive domain scores, using the MSSES, MFIS-5, BDI, MSIS-29, and EDSS scores, gender, and age as independent variables, are presented in Table 3.

Forty percent of Power of Attention variability, 34% of variability in Reaction Time Variability, and 40% of Speed of Memory variability were predicted by total self-efficacy, whereas the other variables' predictive value was evidently lower.

The results of stepwise regression analysis to identify predictors of the self-efficacy scores, using the cognitive domain, MFIS-5, BDI, MSIS-29, and EDSS scores, gender, and age, are presented in Table 4.

Striking findings were the predictive values of disease impact with respect to total self-efficacy (65%) and control self-efficacy (58%) variabilities.

## 4. Discussion

In contrast to the number of studies on the relationship between self-efficacy and physical functions in PwMS, reports on the association between self-efficacy and cognitive performance in these patients are conspicuously absent. This may due to the fact that the conventional disability measure in MS, the EDSS, is biased toward physical disability and poorly assesses cognitive dysfunction and that only recently has cognition received increasing attention from psychological researchers. It has been reported that university students' beliefs about their verbal, numerical, and spatial capabilities correlated with their performance on the respective tests, with *r* values between 0.27 and 0.36. In our CIS and eRRMS study population, the *r* values for the correlations between total self-efficacy and cognitive test performance were between 0.23 and 0.65, and one out of three *r* values was ≥0.49. So it seems that in the early phases of MS the relationship between self-efficacy and cognitive performance may even be stronger than in healthy controls.

Interestingly, two (Power of Attention and Speed of Memory) of the three cognitive domains that we found to have strong correlations with total self-efficacy were among the three domains that were evidently impaired compared to controls. This suggests that in MS self-efficacy may positively influence especially those domains that are affected by the disease. Equally interesting is that some intercorrelations between domain scores were notably stronger in patients than in controls: Power and Continuity of Attention's correlation with Episodic Memory and Speed of Memory with Continuity of Attention and Episodic Memory. Moreover, the intercorrelations between cognitive domain scores that were notably stronger in patients than in controls involved all three of the impaired domains, and, of the five self-efficacy-to-cognition correlations with an *r* value >0.50 and a *P* value <0.01, three did also involve impaired domains (whereas of the unimpaired domains it was only Reaction Time Variability that related to self-efficacy). These observations suggest that the correlations between self-efficacy and cognitive domain performance and the intercorrelations between cognitive domains are not randomly distributed but biased towards relationships that involve impaired domains. We therefore hypothesize that self-efficacy preferentially affects the performance of those cognitive domains that are impaired and that the impairment of a cognitive domain induces other domains to partially compensate. For example, Episodic Memory was involved in stronger-than-normal intercorrelations with Power of Attention, Cognitive Reaction Time, and Speed of Memory. With respect to a possible compensatory role of Episodic Memory we believe that an optimal retrieval of contextual information about events or experiences may help patients with deficient Speed of Memory and Attention to complete tasks or execute challenges relating to executive functions. Our hypothesis also implies that cognitive training in persons with early MS should perhaps not be limited to impaired domains but also should include preventive optimization of intact functions.

PwMS are known to have a lowered self-efficacy. Shnek et al. suggested that it may be the combination of unpredictable disease activity and the possibility of being affected by MS in many different ways that produces a lower self-efficacy [8]. Given the relationship between self-efficacy and cognitive performance found in the present study, it is conceivable that, due to a lowered self-efficacy, persons with MS underperform cognitively even more than is explained by CNS pathology per se. This may have unwanted personal and social consequences, as we previously demonstrated a relationship between Power of Attention and Memory and working hours in these patients [21].

Self-efficacy is an important determinant of physical activity [35] and it is measured and managed actively in education and rehabilitation programs [16, 36]. A controlled study indicated that in PwMS efficacy enhancement results in greater levels of well-being and exertion and a better feeling after exercise [9]. In view of our findings it should also be considered that interventions aiming at promoting self-efficacy may effectively improve cognition in persons with MS. As our patients had been diagnosed with CIS or RRMS only about year before study assessment, self-efficacy increasing measures should perhaps be started early in the disease. In addition, as the total self-efficacy variability was for 65% explained by disease impact, our observations draw attention to the importance of considering early DMD treatment, in order to prevent an increase in disease impact.

It may be hypothesized that cognitive functions positively affect self-efficacy, instead of self-efficacy affecting cognitive performance. Patients with better cognitive function could feel more capable of managing symptoms and coping with the demands of MS than patients with an impaired cognition. The most effective way of creating a strong sense of efficacy is through mastery experiences [7], and prima facie it is not clear how relatively slight changes in Power of Attention, Reaction Time Variability, and Speed of Memory could have an impact on the management of symptoms in patients with low levels of disability. The same holds for other factors contributing to self-efficacy, like vicarious experiences provided by social models and verbal persuasion. Future studies on the relationship between self-efficacy and cognition should investigate this hypothesis.

Our study has several limitations. First, we assessed MS-related self-efficacy, being self-efficacy with respect to functional abilities, management of symptoms, and coping with the demands of MS, and not specifically self-efficacy with respect to cognitive test performance. On the other hand, given the frequency and impact of cognitive impairment in MS, it may be expected that MS-related self-efficacy includes self-efficacy regarding cognitive functioning. Moreover, since we performed the cognitive tests in the context of a formal MS study on cognition and since the participants were aware of the fact that cognition may be impaired in MS, a relationship between MS-related self-efficacy and cognitive test performance is quite conceivable. Second, the analyses were explorative and to prevent relevant associations from going unnoticed we did no correction for multiple testing [37, 38]. However, the research questions on the relationship between self-efficacy and cognitive performance were predefined and in such a setting messages can be derived from patterns of exploratory *P* values or very small *P* values [39]. Our interpretation follows these rules, as we did not try not to overstress or overinterpret the findings and were conservative (*r*≥ 0.50; *P* values < 0.01) when selecting relationships for further interpretations. Third, the cross-sectional design of the study precludes conclusions on causality. However, in view of recent reports [40, 41] a causal relationship between self-efficacy and cognitive performance is most likely. Fourth, our findings do not inform on the relationship between self-efficacy and cognitive functioning in real life settings, as it is unknown how the test results translate themselves in everyday life. Future studies in PwMS on the relationship between MS-related self-efficacy and cognition should therefore include assessments of activities and tasks involving cognition in daily life by using patient-reported outcomes and assessments by proxies.

## 5. Conclusions

Persons with CIS and eRRMS showed moderate to good and highly significant correlations between total self-efficacy and Power of Attention, Reaction Time Variability, and Speed of Memory. On stepwise regression analyses 40% of Power of Attention, 34% of Reaction Time Variability, and 40% of Speed of Memory variabilities were predicted by total self-efficacy. These findings may suggest that in persons with CIS and eRRMS self-efficacy may have a positive impact on cognitive performance, especially in those domains that are affected by the disease, and that prevention of disease activity may preserve self-efficacy.

## Figures and Tables

**(a):** 

	Continuity of Attention	Cognitive Reaction Time	Reaction Time Variability	Working Memory	Episodic Memory	Speed of Memory
Power of Attention (ms)	−0.27	0.56^#^	0.38^+^	−0.19	−0.38^+^	0.78^∧^
Continuity of Attention (#)		−0.18	−0.38^+^	0.25	0.38^+^	−0.41^+^
Cognitive Reaction Time (ms)			0.33	−0.14	0.15	0.44^+^
Reaction Time Variability (CV)				−0.29	−0.01	0.21
Working Memory (SI)					0.3	−0.17
Episodic Memory (#)						−0.4^+^

CIS, Clinically Isolated Syndrome; eRRMS, early Relapsing Remitting multiple sclerosis; ms, milliseconds; CV, coefficient of variance; SI, sensitivity index; ^+^ *P* < 0.05; ^#^ *P* < 0.001; ^∧^ *P* < 0.0001.

**(b):** 

	Continuity of Attention	Cognitive Reaction Time	Reaction Time Variability	Working Memory	Episodic Memory	Speed of Memory
Power of Attention (ms)	−0.03	0.52	0.53	−0.08	−0.06	0.54
Continuity of Attention (#)		−0.07	−0.39	0.14	0.15	−0.03
Cognitive Reaction Time (ms)			0.4	−0.14	−0.06	0.39
Reaction Time Variability (CV)				−0.07	−0.05	0.26
Working Memory (SI)					0.31	−0.22
Episodic Memory (#)						−0.1

ms, milliseconds; CV, coefficient of variance; SI, sensitivity index; correlations were not tested for statistical significance.

**Table 2:** Pearson coefficients for the correlations between self-efficacy and cognitive domain scores in persons with CIS and eRRMS (*N* = 33).

MSSES	Power of Attention	Continuity of Attention	Cognitive Reaction Time	Reaction Time Variability	Working Memory	Episodic Memory	Speed of Memory

MSSES-Function	−0.49^∗^	0.49^∗^	0.09	−0.36^+^	0.14	0.43^+^	−0.51^∗^
MSSES-Control	−0.32	0.17	−0.41^∗^	−0.55^∗^	0.22	0.2	−0.39^∗^
MSSES-Total	−0.65^#^	0.36^∗^	−0.25	−0.57^#^	0.23	0.36^+^	−0.53^∗^

CIS, Clinically Isolated Syndrome; eRRMS, early Relapsing Remitting multiple sclerosis; MSSES, Multiple Sclerosis Self-Efficacy Scale; ^+^ *P* < 0.05; ^∗^ *P* < 0.01; ^#^ *P* < 0.001.

**Table 3:** Multivariate prediction of cognitive domain scores in persons with CIS and eRRMS (*N* = 33).

Measure	Predictors	Partial *R*-square	Model *R*-square

Power of Attention	MSSES-Total	0.4011	0.4011
	Age	0.0500	0.4511

Continuity of Attention	MSSES-Function	0.2245	0.2245
	Age	0.0633	0.2878

Cognitive Reaction Time	MSSES-Control	0.1566	0.1566
	MFIS-5	0.2216	0.3782
	BDI	0.1127	0.4910
	Age	0.1422	0.6332

Reaction Time Variability	MSSES-Total	0.3372	0.3372

Working Memory	Gender	0.1745	0.1745
	MSIS-29	0.1477	0.3223
	Age	0.1284	0.4507
	MSSES-Total	0.0525	0.5031

Episodic Memory	MSSES-Function	0.2109	0.2109
	EDSS	0.1442	0.3551

Speed of Memory	MSSES-Total	0.4011	0.4011
	Age	0.0500	0.4511

CIS, Clinically Isolated Syndrome; eRRMS, early Relapsing Remitting multiple sclerosis; MSSES, Multiple Sclerosis Self-Efficacy Scale; MFIS-5, Modified Fatigue Impact Scale 5-Item Version; BDI, Beck Depression Inventory; MSIS-29, Multiple Sclerosis Impact Scale-29; EDSS, Expanded Disability Status Scale.

**Table 4:** Multivariate prediction of self-efficacy scores in persons with CIS and eRRMS (*N* = 33).

Measure	Predictors	Partial *R*-square	Model *R*-square

MSSES-Total	MSIS-29	0.6495	0.6495
	Power of Attention	0.1098	0.7592
	MFIS-5	0.0382	0.7974
	Age	0.0257	0.8231
	Continuity of Attention	0.0375	0.8606

MSSES-Function	MFIS-5	0.2890	0.2890
	Continuity of Attention	0.1705	0.4595
	EDSS	0.0816	0.5411
	Speed of Memory	0.0704	0.6115
	Cognitive Reaction Time	0.0726	0.6842
	Working Memory	0.0326	0.7168

MSSES-Control	MSIS-29	0.5799	0.5799
	Cognitive Reaction Time	0.0897	0.6696
	BDI	0.0875	0.7571
	MFIS-5	0.0700	0.8271
	MSIS-29	0.0029	0.8242
	Age	0.0615	0.8857

CIS, Clinically Isolated Syndrome; eRRMS, early Relapsing Remitting multiple sclerosis; MSSES, Multiple Sclerosis Self-Efficacy Scale; MSIS-29, Multiple Sclerosis Impact Scale-29; MFIS-5, Modified Fatigue Impact Scale 5-Item Version; EDSS, Expanded Disability Status Scale; BDI, Beck Depression Inventory.
